# The Involvement of Lysosomes in Myocardial Aging and Disease 

**DOI:** 10.2174/157340308784245801

**Published:** 2008-05

**Authors:** Alexei Terman, Tino Kurz, Bertil Gustafsson, Ulf T Brunk

**Affiliations:** 1 Division of Geriatric Medicine, Faculty of Health Sciences, Linköping University, 58185 Linköping, Sweden; 2 Laboratory of Clinical Pathology and Cytology, Karolinska University Hospital, 17178 Stockholm, Sweden; 3 Division of Pharmacology, Faculty of Health Sciences, Linköping University, 58185 Linköping, Sweden; 4 Department of Pathology and Cytology, University Hospital, 58185 Linköping, Sweden

**Keywords:** Aging, apoptosis, autophagy, cardiac myocytes, mitochondria, oxidative stress.

## Abstract

The myocardium is mainly composed of long-lived postmitotic cells with, if there is any at all, a very low rate of replacement through the division and differentiation of stem cells. As a consequence, cardiac myocytes gradually undergo pronounced age-related alterations which, furthermore, occur at a rate that inversely correlates with the longevity of species. Basically, these alterations represent the accumulation of structures that have been damaged by oxidation and that are useless and often harmful. These structures (so-called ‘waste’ materials), include defective mitochondria, aberrant cytosolic proteins, often in aggregated form, and lipofuscin, which is an intralysosomal undegradable polymeric substance. The accumulation of ‘waste’ reflects the insufficient capacity for autophagy of the lysosomal compartment, as well as the less than perfect functioning of proteasomes, calpains and other cellular digestive systems. Senescent mitochondria are usually enlarged, show reduced potential over their inner membrane, are deficient in ATP production, and often produce increased amounts of reactive oxygen species. The turnover of damaged cellular structures is hindered by an increased lipofuscin loading of the lysosomal compartment. This particularly restricts the autophagic turnover of enlarged, defective mitochondria, by diverting the flow of lysosomal hydrolases from autophagic vacuoles to lipofuscin-loaded lysosomes where the enzymes are lost, since lipofuscin is not degradable by lysosomal hydrolases. As a consequence, aged lipofuscin-rich cardiac myocytes become overloaded with damaged mitochondria, leading to increased oxidative stress, apoptotic cell death, and the gradual development of heart failure. Defective lysosomal function also underlies myocardial degeneration in various lysosomal storage diseases, while other forms of cardiomyopathies develop due to mitochondrial DNA mutations, resulting in an accumulation of abnormal mitochondria that are not properly eliminated by autophagy. The degradation of iron-saturated ferritin in lysosomes mediates myocardial injury in hemochromatosis, an acquired or hereditary disease associated with iron overload. Lysosomes then become sensitized to oxidative stress by the overload of low mass, redox-active iron that accumulates when iron-saturated ferritin is degraded following autophagy. Lysosomal destabilization is of importance in the induction and/or execution of programmed cell death (either classical apoptotic or autophagic), which is a common manifestation of myocardial aging and a variety of cardiac pathologies.

## INTRODUCTION

The cardiac muscle is a nonstop working machine that is characterized by a predominantly aerobic metabolism and one of the highest oxygen consumption rates of any organs [1]. Heart myocytes have long been considered irreplaceable, being as old as the whole organism. Only relatively recently, when stem cells with the potential to differentiate into mature myocytes were found even in adult hearts [2], was this view reconsidered. Yet, cardiac myocytes, along with neurons and retinal pigment epithelial cells, are replaced much more slowly than most other tissues and, therefore, are called ‘long-lived postmitotic cells’ [3]. It has been speculated that infrequent cell replacement might be advantageous for certain functions, such as long-term memory in neurons and compensatory hypertrophy in heart myocytes [4]. At any rate, both the intensive function and the low pace of replacement make cardiac myocytes highly vulnerable to damage associated with aging and various diseases [5, 6].

Due to a basic, unavoidable electron leak from the mitochondrial electron transport chain and an imperfect antioxidant defense system (primarily operated by superoxide dismutase, catalase and glutathione peroxidase), normal biological respiration is associated with continuous mild oxidative damage to biomolecules such as nucleic acids, proteins and lipids [7, 8]. This normal damage is not completely repaired and so has a tendency to accumulate over time. Apparently, it is the main contributor to the progressive structural deterioration, reduced function, decreased adaptability, and increased probability of disease and death, i.e., biological aging, or senescence [9, 10]. In a highly aerobic organ such as the heart, the manifestations of aging are extremely pronounced, involving first and foremost the mitochondria and the lysosomes of cardiac myocytes (see below).

Many lines of evidence suggest that a continuous endogenous production of reactive oxygen species (ROS), especially from the mitochondria, is a major contributor to aging [7, 11]. In contrast to senescence, diseases affect particular individuals and are usually more dependent on conditions. They often occur when the effects of damaging (pathogenic) factors exceed the organism’s adaptability. Due to the almost complete lack of repair of the heart muscle through proliferation, both myocardial aging and pathologies (such as ischemic heart disease, metabolic and inflammatory disorders) are associated with the irreversible loss of cells. For the same reason, tumors rarely develop in the myocardium, in contrast to tissues with high proliferative capacity, such as the epithelium of the digestive tract and the bone marrow. Cells of the latter tissues show negligible senescence, but a high incidence of neoplasms, deriving from undifferentiated progenitor cells [12].

Lack of repair through proliferation increases the demands on intracellular renewal mechanisms, which include not only the synthesis of macromolecules and organelle biogenesis, but also involves the systems responsible for removal of damaged and obsolete cellular structures. These systems involve cytosolic proteases, proteasomes and, most importantly, lysosomes, which are responsible for the degradation of virtually all types of intracellular material and a number of signaling pathways, including the regulation of apoptosis. Here we discuss the role of lysosomes in relation to myocardial aging and pathology. 

## LYSOSOMES AND CARDIAC MYOCYTE HOMEOSTASIS

The degradation within lysosomes of each cell’s own constituents is called autophagy, or autophagocytosis [13-15]. Autophagy is an ongoing process, reflecting the need of cells for continuous (basic) renewal. Consequently, it needs to be emphasized that autophagy is a perfectly normal process that is necessary for the normal turnover of organelles and a variety of biomolecules, mostly long-lived proteins. The regulation of, and the different steps involved in autophagy have recently been explored in some detail, mainly through studies on yeast. Dozens of strikingly well conserved autophagy-related proteins and corresponding genes have been discovered and given the name ‘Atg’ [16, 17]. These proteins have been found in various mammalian tissues, including the myocardium [18] The importance of autophagy for normal heart function is demonstrated by the fact that cardiac-specific inhibition of Atg5 results in severe cardiomyopathy in mice [19].

Autophagy, however, may also act as a life-sustaining mechanism under stress conditions by allowing cells to continue vital metabolism by ‘self-eating’ such parts that can be replaced later - in the event the cell survives. This type of autophagy is obviously an attempt of the cell to provide itself with substrates for ATP production and building blocks for essential anabolism by lysosomal degradation of various macromolecular structures [20]. Under starvation, cells in culture are thus found to considerably increase the rate of autophagy [21]. Prevention of autophagy (e.g. by inhibiting the formation of the autophagic membrane by 3-methyl-adenine) or suppressing lysosomal enzyme activity, results in rapid cell death [21, 22]. In starving animals, autophagy is enhanced in the liver and the same is found in the hearts of newborn rats if feeding is withheld [23-25]. 

Autophagy is also activated in response to various stressors associated with abnormal damage to cellular structures, e.g. oxidants, ionizing radiation, hypoxia, etc. This type of autophagy may be named reparative autophagy, since its purpose is to rid the cell of damaged structures and replace them with new ones. In cardiac myocytes, reparative autophagy most commonly occurs under ischemia/reperfusion [26-29] as well as under *in vitro* conditions that imitate such type of stress [30]. Insufficient reparative autophagy may lead to irreversible cell injury when damaged, poorly performing mitochondria accumulate and start to release ROS and pro-apoptotic molecules, culminating in programmed cell death, PCD (see below).

Depending on how material destined for degradation enters lysosomes, three types of autophagy are recognized in mammalian cells: macroautophagy, microautophagy, and chaperone-mediated autophagy (Fig. (**1**)). In macroautophagy, a portion of cytoplasm is enclosed in a double-membrane-bound vacuole termed the autophagosome [13-15]. Precursors of autophagosomes are called phagophores [31] or pre-autophagosomes [32]. They emerge as small vesicles that transform into cup-like structures and then into double-membrane vacuoles (autophagosomes) when the edges of the ‘cups’ fuse. The origin of the autophagosome membrane is still disputed. It was assumed that it might arise from other organelles such as the endoplasmic reticulum or the Golgi complex [31]. Studies on yeast, however, suggested the *de novo* development of such a membrane because it showed a unique protein composition, different from that in other biological membranes [33]. Autophagosomes then fuse with lysosomes or late endosomes, and the engulfed material is degraded by a variety of hydrolytic enzymes. Macroautophagy is the most universal form of autophagy, providing for degradation of almost all types of cellular structures including proteins, lipids, complex carbohydrates, nucleic acids and other biomolecules, as well as various organelles, such as mitochondria, fragments of endoplasmic reticulum, ribosomes, and peroxisomes [3, 15]. Macroautophagy is particularly important for cardiac myocytes whose abundant, actively respiring, mitochondria suffer oxidative damage and need to be efficiently turned over. 

In microautophagy, macromolecules or small organelles are taken up by lysosomes through invagination of the lysosomal membrane, initially resulting in the formation of multivesicular bodies [14]. Microautophagy has been described in various cell types including cardiac myocytes [20]. 

Chaperone-mediated autophagy (CMA) is the selective transport of specific proteins into lysosomes by molecular chaperones. Targeted proteins contain an amino acid sequence KFERQ (lysine-phenylalanine-glutamate-arginine-glutamine) that is recognized by a heat-shock chaperone protein Hsp73. The substrate-chaperone complex then binds to the lysosome-associated membrane protein 2a (LAMP-2a) and enters the lysosome [34-36]. 

## LYSOSOMES AND CELL DEATH

While autophagy is a programmed degradation of altered cellular constituents, PCD is a removal of irreversibly damaged cells. Autophagy usually promotes cell survival, but when cellular damage is pronounced, it can also participate in PCD (see below). Both autophagy and PCD are adaptive processes, working in concert and providing for renewal of the organism’s constituents and life maintenance. PCD, unlike necrosis (accidental cell death), is a form of cell death that involves minimal damage to adjacent cells. It occurs either through classical apoptosis (PCD-I), when cellular constituents are degraded by a family of cytosolic cysteine endopeptidases called caspases, or through autophagic cell death (PCD-II) in which cell parts are autophagocytosed and digested within lysosomes [37, 38]. Often classic, caspase-mediated apoptosis and autophagic cell death are mixed, with dying cells showing both caspase activation and enhanced numbers of autophagosomes [37, 38].

PCD plays a major role in the elimination of unnecessary, damaged, or diseased cells during the whole life span, and is especially prominent during embryogenesis, when a majority of newly formed cells undergo PCD during the development of the individual. Major pathologies, such as cancer and neurodegenerative diseases, are associated with an abnormal depression or enhancement, respectively, of PCD, and apoptotic cell death with a secondary dystrophic calcification seems to be an important factor in the formation of atherosclerotic plaques [39, 40]. 

Although Christian de Duve, who discovered lysosomes in the late 1950s, nicknamed them ‘suicide bags’, lysosomes for a long time were believed to be of importance only for necrotic (accidental) cell death, while true cell suicide, named apoptosis (currently known as PCD-I, see above), was considered an effect of either ligation of death receptors along the plasma membrane (the external pathway) that sets off a cascade of caspase activation, or the release of pro-apoptotic mitochondrial proteins due to apoptogenic stimuli from within the cell (the internal pathway) [41, 42]. It needs to be pointed out that the external pathway usually also affects mitochondria resulting in the release of apoptogenic agonists. Both of these pathways involve the activation of a cascade of caspases and result in a controlled manner of cell death by which the affected cells are fragmented into apoptotic bodies with an intact surrounding plasma membrane. The apoptotic bodies are then phagocytosed by neighboring cells, or by professional scavengers such as macrophages, resulting in a quiet and peaceful elimination of the apoptotic cells without the inflammatory response that is seen in necrosis [43]. Moreover, the apoptotic cells are not wasted but serve as ‘food’ following degradation within the lysosomal compartment of the engulfing cells. 

Several years ago, the finding of lysosomal membrane labilization prior to manifested PCD, especially in oxidative stress-induced apoptosis, in combination with the observation that lysosomotropic detergents, which specifically damage lysosomes, induce classical apoptosis, led to the suggestion that lysosomes were involved in the apoptotic process (reviewed in [44, 45]). However, this idea initially evoked limited enthusiasm among the more doctrinaire cell death experts. Not until it was shown that activation of the pro-apoptotic proteins, Bid and Bax, could be accomplished by lysosomal proteases, did the role of lysosomes in apoptosis become somewhat more widely recognized [46, 47].

Moderate oxidative stress is known to induce classical apoptosis of most cells in culture, but higher levels of such stress are more closely associated with necrosis [44, 48-50]. An upstream event in the apoptotic process induced by oxidative stress was found to be lysosomal rupture due to iron-catalyzed peroxidation of lysosomal membranes that, in turn, was a consequence of the high concentrations of low-mass iron compounds in many lysosomes secondary to autophagic degradation of ferruginous materials, such as mitochondrial respiratory complexes [51-54]. Lysosomal rupture and consequent apoptosis or necrosis following oxidative stress was shown to be a function of the concentration of low-mass iron compounds within lysosomes. Similarly the varied degrees of stability of lysosomes in a single cell are probably a reflection of whether or not the individual lysosome has recently been engaged in autophagy of ferruginous structures [15, 55-57]. It follows that in iron-overload diseases, such as in the various forms of hemochromatosis, when lysosomes are known to be rich in low-mass compounds containing labile iron, the lysosomes, and thereby their host cell as well, are abnormally sensitive to oxidative stress [56, 58-60]. 

Given the importance of keeping the labile redox-active iron content of lysosomes as low as possible in order to minimize the occurrence of rupture and ensuing apoptosis/necrosis, it might be expected that mechanisms to this end have evolved. It seems they have: it has been found that cells with upregulated phase II stress proteins, such as ferritin, metallothioneins and heat shock proteins are particularly resistant to oxidative stress [61-64]. It appears that one of the molecular mechanisms behind this resistance is a continuous autophagic turnover of these proteins that for a limited period of time are able to bind iron within lysosomes in non-redox-active form. 

It may be speculated that excessive levels of autophagy associated with cell repair or starvation (see above) result in rupture of some autophagolysosomes with release of lysosomal hydrolases and ensuing apoptosis. The mechanism behind such rupture may be that the lysosomal compartment is greatly enriched with labile iron when substantial parts of it have been subjected to autophagy, e.g., as a result of degradation of mitochondria containing metalloproteins such as cytochrome *c* (Fig. (**2**)). It can be assumed that even a normal, or only slightly elevated, ROS production would be enough to rupture iron-rich lysosomes [65]. 

As mentioned above, classical apoptosis (PCD-1) is often mixed with autophagic cell death (PCD-2). Lysosomes are involved in both cases, although in PCD-1 lysosomal enzymes mainly participate in the initiation of apoptosis, while in PCD-2 they are the instruments of cell death. The relationship between PCD-1 and PCD-2 follows, in particular, from the fact that Bcl-2 not only inhibits classical apoptosis but also autophagy through suppression of Beclin1 [66]. Conversely, suppression of Bcl-2 would activate both PCD-1 and PCD-2 [18]. Beclin1-mediated autophagy seems to be involved in myocardial degeneration in ischemia/reperfusion injury, while reparative autophagy in mild ischemia is stimulated by an AMP-activated protein kinase [29]. Autophagic degeneration and death of cardiac myocytes has been shown to be a common event in the failing heart [67-70].

## IMPERFECT LYSOSOME FUNCTION IN AGING AND HEART DISEASE

One of the most obvious subcellular characteristics of aging postmitotic cells is a time-dependent accumulation of the age pigment lipofuscin within the lysosomal compartment [71]. This accumulation is inversely related to the life span and, consequently, similar postmitotic cells, including cardiac myocytes, become loaded at very different rates when short-lived animals are compared to long-lived ones [72]. This fact by itself suggests that the accumulation of lipofuscin is a disadvantage for cells and may hamper important functions. Lipofuscin is a yellow-brownish pigment with a broad autofluorescence in response to excitation with ultraviolet, blue or green light [73]. It is a heterogeneous polymer composed of protein fragments linked together by aldehyde bridges, which arises from decomposed lipid-derived peroxides. The formation of lipofuscin demands iron-catalyzed peroxidation of intralysosomal material under decomposition, and autophagocytosed mitochondria seem to be a major source of lipofuscin [74, 75]. 

In cell culture systems, the formation of lipofuscin (or ceroid as the material is often called when accumulated due to processes other than pure aging) can easily be influenced. The addition of purified mitochondria to human astrocyte cultures was found to induce the phagocytosis of these organelles with ensuing transport to the lysosomal compartment and their rapid conversion to lipofuscin-like material, verifying that mitochondria may be an origin of lipofuscin [76]. When neonatal rat cardiac myocytes, or growth-arrested human foreskin fibroblasts, are cultured at 40% ambient oxygen, or in the presence of enhanced amounts of iron in the medium, lipofuscin formation is much accelerated in comparison to standard culture conditions. On the other hand, culture at 8% ambient oxygen dramatically reduces lipofuscin formation as compared to standard conditions [77, 78]. These findings point to the importance of iron-catalyzed intralysosomal oxidation for the formation of lipofuscin and suggest that enhanced or delayed intralysosomal degradation would decrease or increase, respectively, the rate of pigment formation, given the same degree of oxidative stress. Rapid degradation of the precursor material should limit the time available for its oxidation and conversion to lipofuscin, while slow degradation gives more time for the oxidative creation of lipofuscin. The application of inhibitors of lysosomal enzymes has verified this hypothesis by showing that, indeed, the prolongation of intralysosomal degradation substantially increases the formation of lipofuscin per unit time [77]. 

Lipofuscin amasses within aging cardiac myocytes and other long-lived postmitotic cells because it is neither degraded, nor exocytosed to any considerable degree. This follows, in particular, from the facts that cultured neonatal rat cardiac myocytes and growth-inhibited human fibroblasts did not show any reversal of lipofuscin content after removal of factors that initially induced its accumulation, i.e., oxidative stress and protease inhibition [77, 78]. Moreover, lipofuscin was not degraded even when fibroblasts were exposed to amino acid starvation, associated with enhanced intralysosomal degradation [21].

Along with lipofuscin accumulation, reflecting incomplete degradation of autophagocytosed material, myocardial aging is characterized by the accretion of extralysosomal ‘waste’ material, including first of all defective mitochondria that for some reason escape autophagic sequestration. It may be speculated that these mitochondria are poorly recognized for autophagy, but this assumption lacks any credible experimental support. De Grey suggested that mitochondria harboring DNA mutations (due to oxidation or replicative errors) and, therefore, poorly respiring and producing decreased amounts of ROS, experience only slight oxidative damage to their membranes. This might make them less likely to be targeted for autophagy as compared to normal mitochondria and results in their progressive accumulation [79]. Yet, this is still a hypothesis. Our study on neonatal rat cardiac myocytes suggests that enlarged mitochondria (which may appear due to disturbed mitochondrial fission secondary to oxidative damage) are autophagocytosed less efficiently than mitochondria of small sizes, leading to their progressive accumulation with age [80]. This observation may explain the accumulation of so-called ‘giant’ mitochondria in aged cardiac myocytes [81] (Fig. (**3**)). 

There are good reasons to believe that in aged cardiac myocytes and other postmitotic cells, extralysosomal ‘garbage’ accumulation is to a large extent secondary to lipofuscin accumulation. Intralysosomal degradation requires a variety of hydrolytic enzymes that are matured in the trans-Golgi network and then transported away in tiny, coated vesicles to fuse with late endosomes, which then develop into lysosomes.

Cytochemical studies on lipofuscin-loaded lysosomes have shown that such lysosomes contain not only lipofuscin, but also lysosomal enzymes, indicating that they receive their share of newly produced lysosomal enzymes [82]. Considering the large number of lipofuscin-loaded lysosomes in aged postmitotic cells, it is reasonable to assume that a majority of the newly produced lysosomal enzymes end up in lysosomes that are completely filled with lipofuscin. Because lipofuscin is non-degradable, such enzymes would be lost to any meaningful activity [83]. Given that there should be an upper limit to the possible production of lysosomal enzymes, such a dysfunctional allocation of lysosomal enzymes would finally cause a situation when lysosomal digestion would be hampered by lack of degradation capacity. Resulting from a predominant transport of newly produced lysosomal hydrolases to lipofuscin-loaded lysosomes, being a consequence of their large number, lysosomes which have not yet accumulated lipofuscin, would lack sufficient enzymes for optimal digestion. The result would be the accumulation of old and damaged mitochondria and other cellular ‘waste’ products, such as misfolded proteins and various materials clumped together in aggresomes [84]. Indeed, when autophagy is hampered in experiments, it has been found to result in the intracellular accumulation of various waste products [85], while lipofuscin-rich myocardial cells that were aged *in vitro* have been found to show signs of disturbed autophagy and the presence of aged, dysfunctional mitochondria [86].

In support of these ideas are the findings that autophagy is indeed depressed in aged cells [87-89]. The consequence is an accumulation of old and damaged mitochondria with reduced membrane potential and ATP production, but with increased production of superoxide and hydrogen peroxide [90, 91]. Such increased oxidative stress in turn may initiate a cross-talk between mitochondria and the lysosomal compartment where lysosomes are induced to leak lytic enzymes that in turn may further damage mitochondria. Such interplay between lysosomes and mitochondria may finally induce apoptosis due to release of lysosomal hydrolases and truncation of Bid/Bax (see above). This scenario is the basis for our mitochondrial-lysosomal axis theory of postmitotic aging and apoptosis [91].

The question regarding harmful effects of lipofuscin on cellular functions is still somewhat debated. In a recent paper, fibroblasts with elevated quantities of lipofuscin due to prolonged cultivation of cells in 40% ambient oxygen have been found increasingly resistant to complete starvation (exposure to phosphate-buffered saline) [92]. It should be pointed out that cultivation of cells in 40% oxygen (mild oxidative stress) makes them more resistant to oxidative damage that occurs during starvation. Cells with low lipofuscin content (used as controls) were not exposed to 40% oxygen and, therefore, a higher resistance of lipofuscin-rich cells to starvation could rather depend on preconditioning to oxidative stress than on lipofuscin *per se* [93]. Our earlier observations, in which fibroblasts from same culture dishes but with different lipofuscin content were compared, demonstrated decreased resistance of lipofuscin-rich cells to amino acid starvation [21] or acute oxidative stress [94]. 

Progressive damage to mitochondria and lysosomes decreases the adaptability of cardiac myocytes and enhances their sensitivity to injury, which is consistent with a growing incidence of cardiomyopathies and heart failure with advancing age [95, 96]. Heart insufficiency develops much faster if lysosomes or mitochondria are already affected, for example as a result of lysosomal storage diseases or mitochondriopathies. Lysosomal storage diseases, such as the Fabry, Pompe, or Danon diseases, are associated with hereditary defects in genes coding for lysosomal enzymes or other proteins involved in lysosomal degradation, resulting in decreased autophagic turnover and a dramatic accumulation of intralysosomal ‘waste’ material [97-99]. In agreement with the scenario suggested in the mitochondrial-lysosomal axis theory, postmitotic cells affected by lysosomal storage diseases have been shown to contain increased amounts of defective, quasi-senescent, giant mitochondria [100, 101]. In mitochondrial cardiomyopathies, which are associated with mutations in mitochondrial DNA, cardiac myocytes accumulate large numbers of dysfunctional, usually enlarged mitochondria [102]. We speculate that these changes may be associated with insufficient mitochondrial autophagy, as described above (Fig. (**3**)), although this possibility has never been investigated. 

In late hemochromatosis, when myocardial cells are overwhelmed by accumulating iron and most ferritin is almost fully iron-saturated, autophagy exacerbates the situation rather than mitigates it, as autophagy of completely iron-saturated ferritin releases, rather than binds lysosomal redox-active iron. This might explain the often sudden onset of cardiac and liver failure in progressive hemochromatosis when probably most ferritin is iron-saturated. In support of this hypothesis, we recently found that endocytotic uptake of fully iron-saturated ferritin considerably sensitized lysosomes to oxidative stress, while endocytosis of apo-ferritin and non iron-saturated ferritin had the opposite effect (Kurz *et al.* in preparation).

It is well known that aged hearts are more sensitive to oxidative stress, e.g. during ischemia/reperfusion, than young ones [103]. Previously, we showed that lipofuscin-loaded cells in culture were sensitized to oxidative stress, something that was considered an effect of the high iron-content of lipofuscin [94]. In support of this idea, it has recently been shown that the higher sensitivity to ischemia/reperfusion of old hearts compared to young ones can be substantially ameliorated by perfusion with iron-chelators [103]. Reperfusion of ischemic organs causes substantial oxidative stress with resulting lysosomal damage due to intralysosomal peroxidative processes, followed by destabilization of the lysosomal membranes. This can be effectively counteracted by water or lipid-soluble iron-chelators that penetrate into lysosomes and bind their content of low-mass-iron compounds into a non-redox active form [104, 105]. Since it seems that oxidative stress, such as ischemia/reperfusion, damages cells and tissues mainly because of oxidative stress-induced lysosomal injury, with resulting apoptosis/necrosis, we suggest that low-molecular-weight iron chelators, such as salicylaldehyde isonicotinoyl hydrazone (SIH) [105-107] or 1,2-dimethyl-3-hydroxypyridin-4-one (deferiprone, L1, CP20) [108, 109] that easily penetrate cellular membranes should be added to preservation solutions. Then, hopefully, heart, liver, kidney, etc. transplants might survive longer *in vitro* and have improved viability after transplantation. Furthermore, similar chelators could be formulated into therapeutic intravenous solutions for emergency treatment of myocardial infarction, brain ischemia, and brain hemorrhage. Moreover, repeated iron chelation could prevent or slow down formation of undegradable material intralysosomally and might therefore serve as an aging intervention strategy.

## CONCLUSION

Taken together, there seems to be substantial evidence to support the hypothesis that the high accumulation of lipofuscin found in myocardial and other postmitotic cells at the very end of all animals’ lifespans hampers normal autophagy and results in the intracellular concentration of aged and malfunctioning mitochondria, misfolded proteins, and other waste products that together appear to have a destructive effect on the performance and survival of such cells. The high concentration of low-mass-iron that seems to be loosely bound to lipofuscin also induces an increased sensitivity of lipofuscin-loaded lysosomes to oxidative stress.

## Figures and Tables

**Fig. (1):**
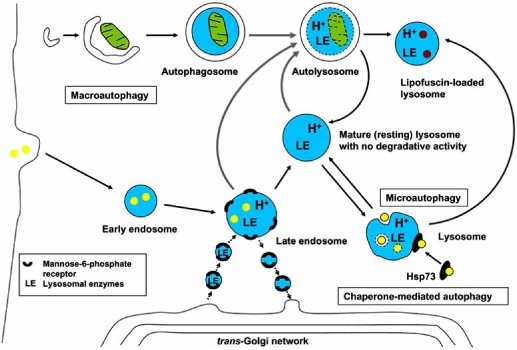
**Autophagy in mammalian cells.** Intracellular material enters lysosomes for degradation through macroautophagy, microautophagy or chaperone-mediated autophagy. Mature lysosomes evolve from late endosomes as a result of their increasing acidification and enrichment with lysosomal enzymes (LE). The latter are delivered (in mannose-6-phosphate receptor-bound form) within specific transport vesicles that are pinched off from the trans-Golgi network. Oxidized, polymerized and undegradable material accumulates within lysosomes in the form of lipofuscin. Lipofuscin-loaded lysosomes maintain an acidic pH and experience permanent influx of lysosomal enzymes. Thin black arrows indicate evolution of structures, thick gray arrows symbolize fusion, while arrowheads show transport processes. References are given in the text.

**Fig. (2):**
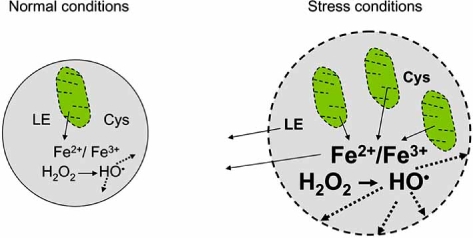
**Tentative mechanism of lysosomal membrane permeabilization secondary to enhanced reparative autophagy.** Reparative autophagy is induced by cellular damage under stress conditions. It is associated with increased number and size of lysosomes (autolysosomes) containing mitochondria and other damaged cellular structures under degradation. This may result in the release of redox-active iron from mitochondrial metalloproteins, activation of Fenton reactions and generation of hydroxyl radicals with ensuing damage to the lysosomal membrane (shown as dashed line). See detailed explanations in the text.

**Fig. (3):**
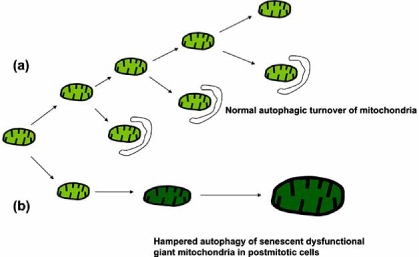
**Tentative mechanism of selective accumulation of giant mitochondria.** Normal turnover of mitochondria is associated with their continuous removal by autophagy and fusion/fission, generating new mitochondria and preventing mitochondrial enlargement (**a**). Mitochondrial damage (e.g., by ROS) may lead to disturbed fission and enlargement of certain mitochondria (**b**). There is a reason to believe that autophagy of enlarged mitochondria is energy consuming and thus more complicated than that of normal mitochondria. Because of this, a further mitochondrial enlargement and a progressive amassing of giant dysfunctional mitochondria may take place within long-lived postmitotic cells, such as cardiac myocytes.
